# A Retrospective Analysis of Late Open Conversions Following Failed Endovascular Aneurysm Repair

**DOI:** 10.31083/j.rcm2510363

**Published:** 2024-10-10

**Authors:** Bahadır Aytekin, Bekir Boğaçhan Akkaya, Hayrettin Levent Mavioğlu, Hakkı Zafer İşcan

**Affiliations:** ^1^Department of Cardiovascular Surgery, Ankara Bilkent City Hospital, 06800 Ankara, Turkey

**Keywords:** late open surgical conversion, failed EVAR

## Abstract

**Background:**

The incidence of late open surgical conversions (OSCs) has recently increased. Vascular surgeons face additional technical challenges in late conversion surgery of failed endovascular aneurysm repair (EVAR) due to the presence of a previously deployed endograft. Based on our institutional experience, this study aimed to delineate methods to improve late open conversion outcomes, proposing solutions for technical challenges.

**Methods:**

All preoperative OSC data on failed EVARs operated in our Cardiovascular Surgery Clinic between January 2017 and January 2024 were evaluated retrospectively. Study endpoints included early (30-day or in-hospital) and late follow-up outcomes. Early outcomes included perioperative mortality and morbidities, intensive care unit (ICU) period, and length of hospital stay (LOS). The main outcome of interest during follow-up was overall survival.

**Results:**

Sixteen patients in our hospital, comprising eight elective and eight emergency procedures, underwent OSCs following EVAR. The difference between the 30-day mortality rates for the elective and urgent late conversions was significant (*p* < 0.001). Of these patients, 15 were male, with a mean age of 70.8 years (range: 62–80). Preoperative cardiac shock status and low hematocrit level (<20%) were independent mortality factors (*p* < 0.001). The ICU period was 8.7 ± 5.3 days (2–20 days) on average, and LOS was 17.3 ± 8.4 (6–29 days) days on average. The mean time to open surgical conversion in this cohort was 44.4 ± 16.8 months. The 5-year overall survival rate was 43.75%.

**Conclusions:**

The incidence of open surgical conversion is notably growing. Emergent open surgical conversions exhibit poorer mortality outcomes compared to elective procedures. Further data are essential to evaluate the ramifications of expanding the use of EVAR beyond the instructions for use (IFU) guidelines. The procedures involving patients who challenge the IFU criteria should be conducted at experienced centers and require close monitoring. Open surgical repair (OSR) as the initial treatment opportunity could be an alternative strategy for improving outcomes in this patient cohort.

## 1. Introduction

Endovascular aneurysm repair (EVAR) has emerged as the preferred treatment modality for infrarenal abdominal aortic aneurysms (AAAs) owing to its less invasive nature and early success [1]. Consequently, 70%–80% of infrarenal AAAs are currently operated in an endovascular manner [2, 3]. This preference is primarily attributable to the demonstrated advantages of EVAR over short to mid-term morbidity and mortality compared to open surgical repair (OSR), especially in frail patient groups [4]. The long-term durability of EVAR is challenged by graft or aneurysm-related complications such as endoleaks, migration, or sac expansion. Randomized control trials have reported no survival benefit in the long term and subsequently recommended mandatory lifelong surveillance [5, 6, 7].

Nearly 30% of patients may require reintervention within 10 years following their initial EVAR [8]. While most interventions can typically be performed using advanced endovascular techniques, such as the deployment of proximal or distal extensions, device relining, use of the Heli-FX EndoAnchor system (Medtronic Vascular, Santa Rosa, CA, USA), and coil or glue embolization of endoleaks, there are instances where a conversion to OSR remains necessary. Notably, such conversions have increased in recent years [9, 10, 11, 12].

Late open surgical conversion (OSC) following EVAR is reserved for sac expansion after reinterventions or untreatable complications by endovascular techniques. However, we must remember that these patients were deemed ineligible for open repair during the initial EVAR treatment. In late conversion surgery after failed EVAR, vascular surgeons face additional technical challenges due to the presence of a previously deployed endograft. Consequently, the decision to proceed with late OSC must be carefully weighed against the associated risks; however, finding another solution is sometimes impossible. Therefore, comprehensive modern real-world evidence is necessary to better understand the risk factors and outcomes related to OSC after failed EVAR [13]. Mortality in this patient cohort has changed over time. Earlier studies reflecting initial experiences with surgical conversions reported a mortality rate of nearly 40%, attributable to the learning curve associated with EVAR. In contrast, more recent literature indicates a mortality of 2.3%. These outcome variations underscore the significant impact of the surgeons’ experiences and technological advancements on reducing mortality rates [14, 15, 16, 17]. Providing early advantages in endovascular procedures may create the risk of mortal conversion surgeries. Thus, using this approach will increase unsolvable patient complications. Therefore, it is important to explore factors leading to poorer outcomes, such as aortic neck anatomy, patency of side branches, or special endograft complications [18, 19, 20].

Our current study emphasized the operators’ instructions for use (IFU) adherence and the critical technical details for patient survival. Additionally, this research could potentially guide improvements in patient selection and surgical techniques, enhancing overall clinical outcomes. Based on our institutional experience, this study aimed to delineate methods to improve late open conversion outcomes and solve technical challenges.

## 2. Materials and Methods

Our retrospective study explored the outcome of infrarenal AAA patients who underwent OSR after failed EVAR from 2017 onwards. The local ethics committee approved the study protocol (E1-21-1724) (01.12.2021). Index EVAR procedure, date, and place of the procedures were noted. Baseline patient characteristics and operative variables of patients undergoing initial EVAR at other national medical centers were obtained from the E-nabiz national database, a program the Ministry of Health prepared to inform users about personal data.

Inclusion criteria were an aortic endograft explantation (total or partial), arterial reconstruction (anatomic or extra-anatomic) with transabdominal or retroperitoneal incision, and surgery performed at least 30 days after the initial EVAR.

A failed EVAR was defined as the need for open conversion due to various complications such as endoleaks, migration, or graft infection at least 30 days after the index EVAR discharge. The interval to open conversion was calculated as the time between the initial EVAR and OSC.

Data were collected on patient demographics (age, gender), initial EVAR date, OSC date, indication for OSC, OSC technique (endograft preservation, explant), clamp position (supraceliac, supra superior mesenteric, suprarenal, infrarenal), intensive care unit (ICU) period, hospital length of stay (LOS), postoperative complications, intraoperative mortality, 30-day postoperative mortality, and long-term postoperative mortality.

As a part of the EVAR surveillance program at our institution, all patients underwent color Doppler ultrasound (CDUS) and computed tomographic angiography (CTA) surveillance at 1, 6, and 12 months. After 12 months, the CTA surveillance was reduced to once annually, but regular CDUS surveillance was continued every 6 months [19]. In cases of type 1 and 3 endoleaks, or enlargement of maximum aneurysm diameter was detected in CDUS, and immediate CTA was performed. After the late OCS, CTA was performed in the first three months after discharge. If no complication was noted, the CTA controls were performed according to patients’ complaints.

### 2.1 Surgical Technique and Details

All patients underwent OSC via either a median abdominal incision or a retroperitoneal approach based on the patient’s clinical condition, aortic cross-clamping requirements, and surgeon’s preference. The retroperitoneal approach was preferred in most cases. Conversely, the transperitoneal approach was selected in emergency scenarios. Proximal bleeding was controlled using a cross-clamp and a Foley catheter to maintain hemodynamic stability. Depending on the urgency of the case, whether free rupture, contained rupture, or hemodynamic instability, either supraceliac or suprarenal clamping was employed for grafts with suprarenal fixation. After stabilizing the hemodynamics and following graft removal, the cross-clamp was moved to the infrarenal region. When infrarenal clamping was not feasible, renal protection was achieved using Ringer’s lactate solution. The proximal anastomosis was performed as expeditiously as possible, with the cross-clamp repositioned to the infrarenal region.

The removal of endografts varied according to the graft type. Partial destruction was applied solely to polyester endografts. In the case of polyester endograft with suprarenal fixation, the graft was sectioned at the anastomosis line in the infrarenal region, leaving the upper segment in place, and proximal anastomosis was subsequently performed at the cut site. Due to their unsuitability for anastomosis, expanded polytetrafluoroethylene (ePTFE) grafts were completely extirpated, with grafts featuring suprarenal extensions were removed by cutting the stent struts with wire cutters. Complete removal, debridement, and rinsing with antibiotics were conducted for graft infections, followed by tube graft interposition. Dacron grafts (InterGard; Intervascular, La Ciotat, France; FlowNit Bioseal, Knitted Polyester Vascular Graft, JOTEC Vascular Prosthesis, JOTEC GmbH, Hechingen, Germany) were employed for all reconstructive procedures.

Study endpoints included early (30-day or in-hospital) and late follow-up outcomes. Early outcomes included perioperative mortality and morbidities, ICU period, and LOS. The primary outcome of interest during follow-up was overall survival.

### 2.2 Statistical Analysis

Normally distributed continuous variables were expressed as mean values ± standard deviation (SD). Categorical variables were expressed as numbers and percentages. The predisposing factors on overall mortality were explored by using univariate regression analysis. Categorical factors were compared using the chi-square or Fisher exact tests. Here, Fisher’s exact test was used to compare early mortality rates between the elective and urgent groups due to the small sample sizes and categorical nature of the data. Kaplan–Meier analysis and log-rank test were used to express survival outcomes and event-free survival and to compare survival curves among different groups. A *p*-value < 0.05 was considered statistically significant. All statistical analyses were performed using the SPSS statistical software (SPSS for Windows 15.0, Inc., Chicago, IL, USA).

## 3. Results

Since 2017, 16 patients in our hospital have undergone OSC following EVAR: eight elective procedures and eight emergencies. Furthermore, 15 (93.7%) of these patients were male, with a mean age of 70.8 years (range: 62–80). Patient comorbidities are detailed in Table 1.

**Table 1.** **Demographics of patients**.

Associated comorbidity	Number of patients (%)
Chronic obstructive pulmonary disease	3 (18.75%)
Chronic renal insufficiency	5 (31.25%)
Coronary artery disease	9 (56.25%)
Diabetes mellitus	2 (12.5%)
Hyperlipidemia	4 (25%)
Hypertension	12 (75%)
Peripheral artery disease	1 (6.25%)

The overall EVAR conversion rate did not accurately represent the rate observed at our institution since 14 out of 16 patients had their initial EVAR procedures performed at other national medical centers. There was no early open conversion. All patients except two were symptomatic with abdominal pain. Regarding the anatomical perspective of exploring CTA images, most patients were outside the IFU criteria. Table 2 shows the factors outside the IFU criteria.

**Table 2.** **Incompatible IFU criterias**.

	Number of patients (%)
Presence of preoperative CTA at the initial EVAR	12 (75%)
Insufficient neck length (<15 mm)	10 (83.3%)
Large proximal aortic neck diameter (>32 mm)	2 (16.6%)
Thrombus and calcification at the aortic neck (>25%)	4 (33.3%)
Aortic neck angulation (>60°)	2 (16.6%)
Combination of factors	8 (66.6%)

EVAR, endovascular aneurysm repair; CTA, computed tomographic angiography; IFU, instructions for use.

Preoperative CTAs of initial EVAR procedures in 12 patients were evaluated using the E-nabiz database. The indications for OSC were endoleaks in 11 cases (type 1 endoleaks in six cases and type 3 endoleaks in four cases, combined type 1 and 2 endoleaks in one case) (68.75%), stent migration in four cases (25%) and stent graft infection in one case (6.25%). The rupture causes were identified: Type 3 endoleaks in two cases, type 1a endoleaks in five cases, and a combination of type 1 and type 2 endoleaks in one case. Rupture cases were managed through emergency surgery.

The overall hospital mortality rate was 43.7% and was particularly high among patients presenting with rupturing. The early mortality rate for patients who underwent elective surgery was 12.5% or 1 in 8 patients. In contrast, the early mortality rate for patients who underwent urgent surgery was 75%. The difference between the 30-day mortality rates for the elective and urgent late conversions was significant (*p* < 0.001). One ruptured patient entered the operation with cardiac resuscitation, while two ruptured patients also had intraoperative cardiac resuscitation. One patient died on the second postoperative day due to multiorgan failure (MOF), while another who experienced cardiac, renal, and pulmonary complications died of MOF on the 20th postoperative day. One out of three patients with intraoperative cardiac resuscitation survived the operation and was successfully discharged. This patient had an elective endovascular intervention for an iliac artery aneurysm three years later and died because of colon cancer in the sixth postoperative year. The graft infection case was a lung cancer patient who had an interventional procedure at the 36th-month post-EVAR in a foreign medical center. He survived the operation; however, he died from sepsis on the fourth postoperative day. There were two cardiac deaths in the early period. Meanwhile, preoperative cardiac shock status and low hematocrit level (<20 %) were independent mortality factors (*p* < 0.001). Table 3 shows the univariate analysis for early mortality.

**Table 3.** **Univariate analysis for early mortality**.

	Number of patients (%)	Hazard ratio (HR)	95% CI	*p*
Coronary artery disease	7 (43.75%)	1.20	0.54–2.65	0.34
Chronic renal insufficiency	4 (25%)	2.40	0.88–6.55	0.09
Hypertension	12 (75%)	0.83	0.43–1.59	0.56
Chronic obstructive pulmonary disease	5 (31.25%)	1.25	0.43–3.63	0.73
Diabetes mellitus	2 (12.5%)	0.67	0.13–3.47	0.8
Hyperlipidemia	6 (37.5%)	0.75	0.15–3.77	0.78
Suprarenal fixation	12 (75%)	2.60	0.91–7.41	0.08
Suprarenal aortic cross-clamping	10 (62.5%)	3.33	1.13–9.82	0.03
Endovascular reinterventions after initial EVAR	9 (56.25)	2.78	0.85–9.08	0.1
Ruptured aneurysm	8 (50%)	7.00	2.07–23.66	0.001
Hemorrhagic shock	6 (37.5%)	6.00	1.79–20.08	0.001
Hematocrit level <20%	5 (31.25%)	5.00	1.49–16.75	0.001

CI, confidence interval; EVAR, endovascular aneurysm repair.

**Fig. 1.**
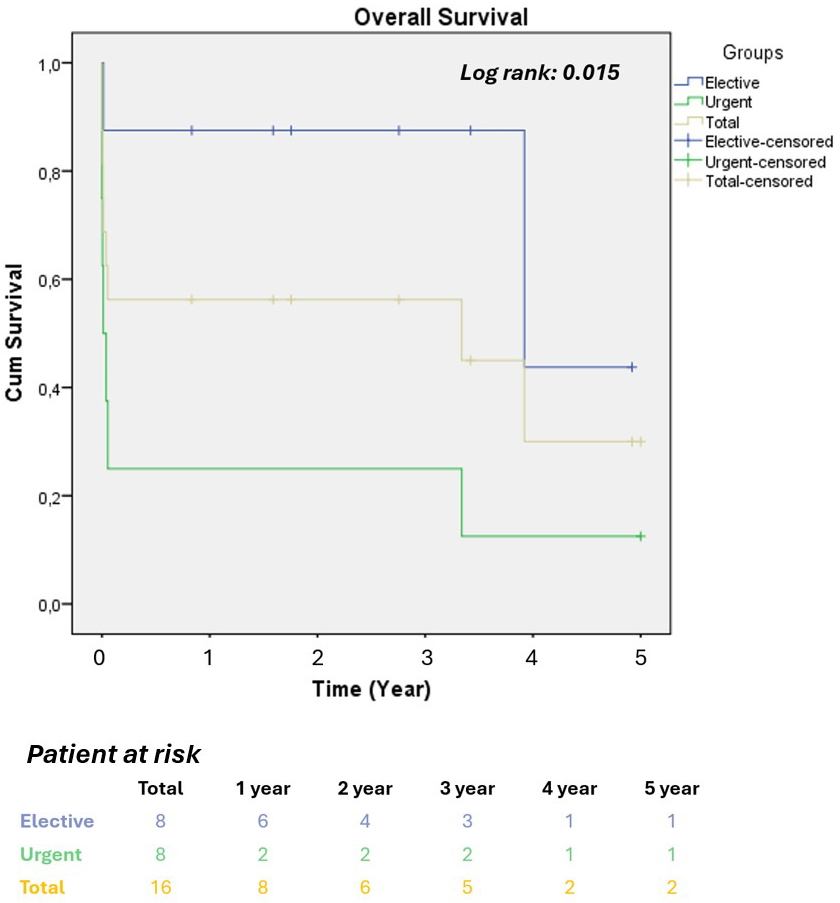
**Kaplan–Meier survival analysis**. The 5-year cumulative mortality rates and number at risk for emergency and elective cases. Statistical differences were analyzed using Kaplan–Meier survival curves and the log-rank test.

The average ICU period was 8.7 ± 5.3 days (2–20 days), and LOS was 17.3 ± 8.4 (6–29 days). The mean time to open surgical conversion in this cohort was 44.4 ± 16.8 months. In the follow-up period, there was one case of cardiac mortality and one instance of rehospitalization due to a suspected graft infection, which subsequently resulted in the patient dying. Another patient was successfully discharged after receiving antibiotic treatment. Another patient had an incisional hernia. One patient had endovascularly treated common iliac aneurysm at the postoperative 36th month. The mean follow-up was 34.9 ± 11.7 months. Kaplan–Meier survival curves were used to compare overall survival between patients with ruptured aneurysms and those with elective aneurysms. The number of patients at risk was assessed in years for both groups (Fig. 1). The overall survival estimated by the Kaplan–Meier analysis was 43.75% at 5 years.

The partial destruction was performed in only polyester endografts (6 patients, 37.5%). A cross-clamp was fitted in 14 cases. The remaining cases were operated on by inserting and controlling the proximal hemorrhage site with a Foley catheter (suprarenal position). Aorto-bi-iliac grafts were used in six patients, with complete removal of the EVAR devices. The endograft types are demonstrated in Table 4.

**Table 4.** **Endograft types**.

Endografts	Number of patients (%)
Medtronic Endurant II (Medtronic, Minneapolis, MN, USA)	5 (31.25%)
AFX Endologix (Endologix, Irvine, CA, USA)	4 (25%)
Jotec (JOTEC, Hechingen, Germany)	3 (18.75%)
Anaconda (Vascutek, Ltd., Inchinnan, UK)	1 (6.25%)
Lifetech Ankura (Shenzhen, China)	2 (12.5%)
Gore Excluder (W. L. Gore & Associates, Flagstaff, AZ, USA)	1 (6.25%)

Aortic tube grafts were used for eight patients. In two patients, an aortofemoral bypass was performed. One patient also underwent a femoropopliteal bypass. Migrated endografts and two endografts with no active fixation system were more easily extirpated. There was no severe injury at the proximal site, and two supraceliac and six suprarenal aortic cross-clamping (XCl) procedures were performed; no renal protection was used for suprarenal clamping. The encountered postoperative complications are presented in Table 5.

**Table 5.** **Postoperative complications**.

Postoperative complications	Elective OSR	Urgent OSR
	Number of patients (%)	Number of patients (%)
Intraoperative death	0	2 (25%)
Revision due to hemorrhage	0	4 (50%)
Pulmonary	1 (12.5%)	3 (37%)
Cardiac	0	1 (12.5%)
Renal	1 (12.5%)	3 (50%)
Infection	1 (12.5%)	1 (12.5%)
Cerebrovascular event	0	1 (12.5%)
Limb ischemia	0	1 (12.5%)
Multiorgan failure	0	2 (25%)

OSR, open surgical repair.

**Fig. 2.**
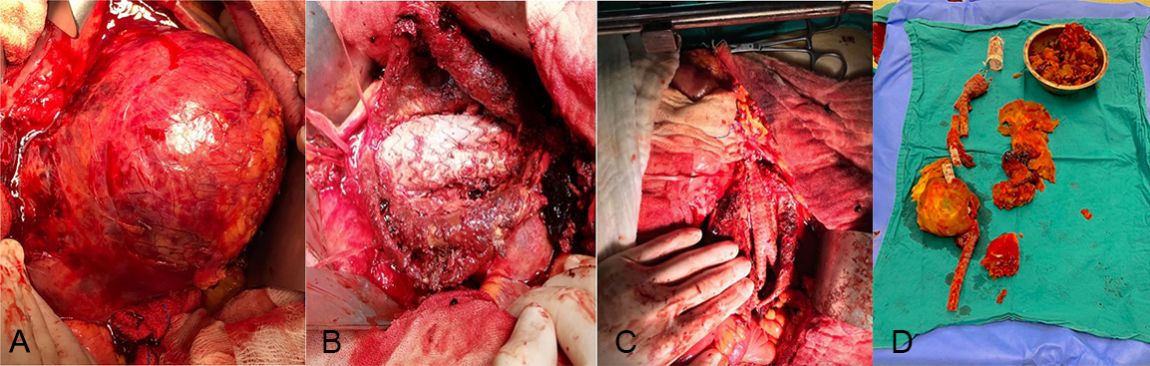
**Open repair of a patient with type 1a endoleak after EVAR**. (A) A patient experiencing aneurysm sac expansion due to type 1a endoleak with aortic cuff treatment, (B) the endograft, (C) late open conversion in the elective manner, and (D) the extirpated endograft. EVAR, endovascular aneurysm repair.

**Fig. 3.**
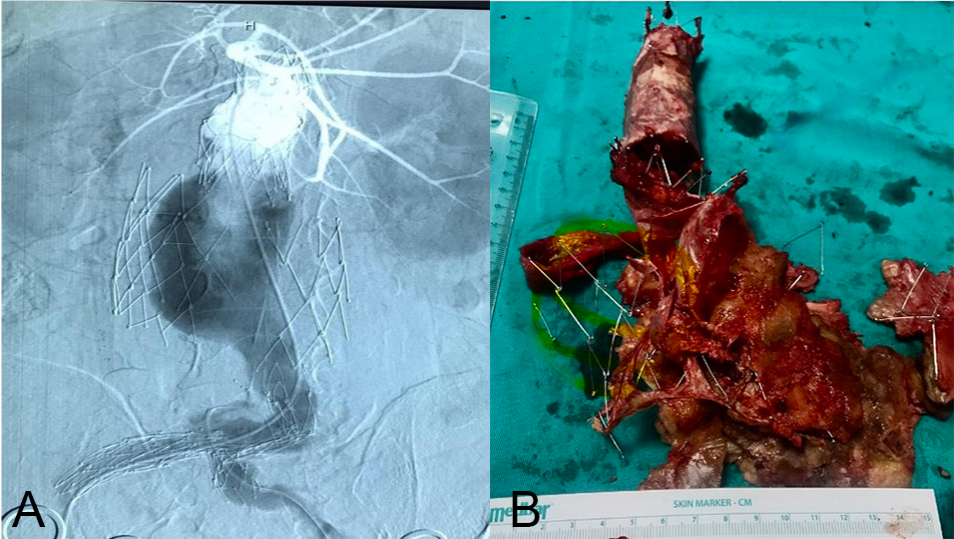
**Open repair of a patient with type 3 endoleak after EVAR**. (A) The angiography image of the patient with type 3 endoleaks. The expansion of the endoskeleton and the issue with the main body deployment over the guidewire. (B) The separated Endologix AFX (Irvine, CA, USA) endograft was extirpated through open surgery. EVAR, endovascular aneurysm repair.

Fig. 2A–C shows a patient experiencing type 1a endoleak who had aortic cuff treatment and finally late open conversion in an elective manner. Fig. 2D shows the extirpated endografts in the patient. Fig. 3A,B shows a patient with type 3 endoleaks and rupturing.

## 4. Discussion

The literature on mortality rates associated with late OSC following EVAR demonstrates considerable variability. Kouvelos *et al*. [21] reported a 30-day overall mortality rate of 9.1%, with a notable disparity between elective and non-elective procedures: 3.2% and 29.2%, respectively. Further, Rinaldi *et al*. [12] found a 30-day mortality rate of 3.3%. In contrast, Yei *et al*. [3] documented a 6-year mortality rate of 35.6%. This range in mortality rates highlights the complexity of outcomes associated with these procedures and underscores the influence of factors such as the nature of the procedure (elective *vs*. non-elective) and the follow-up duration on mortality statistics [14, 17]. In our study, the early mortality rate for emergency cases was 75%, whereas in elective cases, it was 12.5%; however, it should be noted that mortality was observed in only one patient. The early mortality for the total patient cohort was 43.7%. Although our mortality rates may seem elevated compared to some in previous studies, this increased rate can be attributed to the high proportion of emergency cases. As reported in the literature, late conversions from EVAR to OSC are technically feasible but associated with significant morbidity and mortality rates, particularly when performed for ruptures. The complexity of the procedure and the critical condition of the patient contribute to these elevated rates of adverse outcomes [17, 22, 23, 24].

The indications for OSC in our cohort were endoleaks (68.75%), stent graft migration (25%), and infection, which accounted for one case (6.25%). Eight cases presented ruptured aneurysms. These findings underscore the critical importance of vigilant surveillance and prompt intervention in managing these complex cases. Aneurysm rupturing is correlated with low surveillance control [25]; thus, follow-ups on sac regression are vital, meaning post-EVAR surveillance is mandatory. After evaluating the E-Nabiz national database, six patients (42.9%) from foreign medical centers were found to have no post-EVAR CTA control; 87.5% of the patient cohort was from foreign medical centers that did not have OSR opportunities. Another vital point is that both treatment modalities should be given in a medical center; patients would be pushed to endovascular opportunities outside the IFU criteria if there is no treatment of choice. We have no information on the leading clinics that performed the initial EVAR, such as whether the procedure was conducted in a cardiovascular surgery, interventional radiology, or cardiology clinic. We analyzed preoperative CTA images of 10 patients at the initial EVAR procedure, including ours, and discovered that 91.7% of patients were outside the IFU criteria. Table 2 shows the compatibility of patients according to the IFU criteria.

Previous reinterventions after initial EVAR with aortic cuffs, coil or onyx embolization, and endograft interpositions were present in nine patients. Post-EVAR reinterventions before the late open conversion were not a significant mortality factor. The patients should be directed to open surgery conversion in experienced centers to avoid losing time after the first or second endovascular reinterventions [22].

Certainly, endografts possess unique complications, the most notable being observed as type 3 separations when using the Endologix AFX [20]. While attempts are initially made for an endovascular solution, open surgery remains the only recourse in adverse situations. Additionally, older generation endografts lacking active fixation systems, such as barbs and hooks, exhibit migration with accompanying type 1 endoleaks.

Postoperative care, including monitoring for complications such as bleeding, infection, cardiac events, and renal dysfunction, is essential for mitigating risks and optimizing outcomes. The experience and expertise of the surgical team in managing complex vascular procedures and complications associated with EVAR and OSC play a significant role in determining surgical outcomes. The hospital case volume and experience are crucial parameters. While there are no restrictions for emergency cases, there should be a mandated annual number of OSRs performed for elective EVAR procedures, as recommended in guidelines [26]. This requirement can also be referenced in terms of reimbursement conditions.

Surgical techniques employed during OSC varied based on individual patient anatomy and the specific complication necessitating conversion. Furthermore, endograft removal procedures differ depending on the type of graft involved. For a polyester endograft with suprarenal fixation, the graft is severed at the anastomosis line in the infrarenal area, leaving the upper portion *in situ*, and the proximal anastomosis is performed on the cut area. In contrast, ePTFE grafts, unsuitable for anastomosis, are removed by cutting the stent struts with wire cutters.

The urgency of a specific case, such as for a free rupture, contained rupture, or hemodynamic instability, dictates the clamping preference. We typically opt for supraceliac or suprarenal clamping for grafts with suprarenal fixation. Once hemodynamic control is achieved, we move the cross-clamp to the infrarenal region following graft removal. If this is not feasible, renal protection is provided using Ringer’s lactate solution, and the proximal anastomosis is completed as rapidly as possible before shifting the cross-clamp to the infrarenal region.

Operative strategies should demonstrate flexibility regarding the approach (transabdominal *vs*. retroperitoneal), cross-clamp position (suprarenal *vs*. infrarenal), and the extent of endograft explantation (partial or complete). The transperitoneal approach is favorable in emergencies because it provides rapid and direct access to internal organs and major vessels. This approach offers an expanded field of view for the abdominal organs and vessels, facilitating improved access to complex structures such as the right iliac artery aneurysm or renal arteries. Additionally, the transperitoneal approach enhances the management of abdominal hemorrhage by allowing for more effective identification and control of bleeding sources. This capability is particularly critical in emergencies. Furthermore, the transperitoneal approach simplifies the implementation of supraceliac or suprarenal clamping techniques for grafts with suprarenal fixation, thereby enhancing the control of hemorrhage. The decision on the degree of endograft excision typically hinges on factors such as the pre-existing device seal, suprarenal fixation struts, and underlying graft infection. Complete removal of an endograft with suprarenal fixation carries risks of aortic, renal, or visceral artery injury, as well as prolonged suprarenal cross-clamp time, all of which contribute to poorer outcomes. Various techniques have been employed to remove suprarenal stents, including circumferential release of barbs from the main body using a wire cutter or employing a 20 mL syringe as a sheath to collapse the suprarenal component [15, 24]. As documented in a meta-analysis by Esposito *et al*. [23], semi-conversions are associated with acceptable 30-day mortality rates and may serve as a viable alternative to full or partial graft explantation in patients for whom aortic cross-clamping is suboptimal. However, graft preservation techniques could not be used in our cohort. Despite the initially high complication rates, we opted for endograft explantation whenever feasible. Both complete and partial endograft explantation are technically complex procedures but can be conducted safely alongside sufficient expertise. Finally, optimal outcomes are consistently achieved when these procedures are performed electively rather than under emergent conditions.

The literature includes numerous small series detailing open conversions following endograft failure, which contribute minimally to our understanding [12, 13]. Lopez Espada *et al*. [22] analyzed data from 348 cases via the VASCUNET international collaboration involving 55 units across 17 countries, noting an increasing incidence of EVARs necessitating open conversion. This observation prompted an inquiry into whether this trend reflects improved survival, prolonging the time frame until endograft failure. The authors also considered cases involving adjunctive treatments, such as lumbar vessel clipping or aneurysm neck banding, aimed at preserving the endograft rather than a full explanation; these interventions accounted for 10% of cases. It could be debated that excluding cases involving less surgical intervention than those requiring graft removal might have provided a more precise delineation. In our cohort, there was no possibility of using graft preservation techniques, cerclages, aortic wrapping, or side branch clipping. While OSC following failed EVAR offers a critical therapeutic option, it is associated with substantial surgical risks, particularly in emergent cases and among older, frail patients with significant comorbidities. The decision to proceed with OSC should be carefully weighed against the potential benefits and risks, with comprehensive preoperative assessment and meticulous perioperative management essential to optimizing outcomes. Our study reinforces the importance of comprehensive preoperative assessment, meticulous surgical planning, and postoperative management strategies in optimizing outcomes for patients requiring OSC post-EVAR.

Nevertheless, open surgical conversion remains a critical option when endograft procedures fail. It is imperative to emphasize the importance of rigorous postoperative surveillance and to prioritize advancements in endovascular technology to minimize reliance on open surgical conversion. Patients who do not undergo meticulous postoperative monitoring, despite having initially been candidates for endovascular interventions, may ultimately present with ruptures and require emergency intervention. Although the conversion frequency to open surgery is relatively low, urgent surgical procedures could be necessitated in such scenarios; urgent open surgical conversions are associated with higher mortality rates compared to elective cases. Endoleaks with secondary sac expansion were our main indication for OSC, and suprarenal aortic cross-clamping was frequently required. Endograft infection and emergent treatment remained associated with poorer short-term survival.

Our study reveals a markedly low adherence to the IFU for the initial EVAR; moreover, there remains no clear consensus among vascular surgeons on this issue. In the study examining the attitudes of Italian vascular surgeons regarding this issue, consensus was reached on 46% of the proposed statements [1]. In “real-world” clinical practice, up to 44% of EVAR procedures are performed outside the IFU, yet these procedures demonstrate acceptable short- and mid-term outcomes. The Delphi methodology appears to corroborate the gap between guideline recommendations and actual clinical practice [1].

The limitations of our study include its retrospective design and relatively small sample size, which may limit generalizability. Furthermore, the survival estimates should be interpreted with caution due to the small sample size and limited number of patients at risk, which may potentially impact the reliability and generalizability of the findings. Additionally, most patients had their initial EVAR procedure in other national medical centers. Preoperative data were acquired from the national e-Nabiz database alongside patients and their relatives.

## 5. Conclusions

The incidence of open surgical conversion is notably growing. Emergent open surgical conversions exhibit poorer mortality outcomes compared to elective procedures. Adherence to the IFU is an important point for EVAR. Further data are essential to evaluate the ramifications of expanding the use of EVAR beyond the IFU guidelines. The procedures involving patients who challenge the IFU criteria should be conducted at experienced centers and require close monitoring. OSR at the initial treatment could be an alternative strategy for improving outcomes in this patient cohort. Future research should focus on refining patient selection criteria, optimizing surgical techniques, and exploring adjunctive endovascular therapies.

## Availability of Data and Materials

The datasets used and/or analyzed during the current study are available from the corresponding author on reasonable request.
